# Opportunities for Treg cell therapy for the treatment of human disease

**DOI:** 10.3389/fimmu.2023.1166135

**Published:** 2023-04-19

**Authors:** Jeffrey A. Bluestone, Brent S. McKenzie, Joshua Beilke, Fred Ramsdell

**Affiliations:** Sonoma Biotherapeutics, South San Francisco, CA, United States

**Keywords:** Treg, immune tolerance, Foxp3, autoimmunity, immune regulation

## Abstract

Regulatory T (Treg) cells are essential for maintaining peripheral tolerance, preventing autoimmunity, and limiting chronic inflammatory diseases. This small CD4+ T cell population can develop in the thymus and in the peripheral tissues of the immune system through the expression of an epigenetically stabilized transcription factor, FOXP3. Treg cells mediate their tolerogenic effects using multiple modes of action, including the production of inhibitory cytokines, cytokine starvation of T effector (*e*.*g*., IL-2), Teff suppression by metabolic disruption, and modulation of antigen-presenting cell maturation or function. These activities together result in the broad control of various immune cell subsets, leading to the suppression of cell activation/expansion and effector functions. Moreover, these cells can facilitate tissue repair to complement their suppressive effects. In recent years, there has been an effort to harness Treg cells as a new therapeutic approach to treat autoimmune and other immunological diseases and, importantly, to re-establish tolerance. Recent synthetic biological advances have enabled the cells to be genetically engineered to achieve tolerance and antigen-specific immune suppression by increasing their specific activity, stability, and efficacy. These cells are now being tested in clinical trials. In this review, we highlight both the advances and the challenges in this arena, focusing on the efforts to develop this new pillar of medicine to treat and cure a variety of diseases.

## 1 History and biology of T regulatory cells

The demonstration of immune tolerance dates to Owens, Burnet, Medawar, and others in the 1950s, including the awarding of a Nobel Prize in 1960 (reviewed in (1). Importantly, over the next two decades, tolerance was shown to be transferable with leukocytes from tolerant animals, suggesting that a specialized cell population was responsible. Although the concept of suppressive T cells was around for many decades starting with the work of Kondo and Gershon (2), the field was littered with phenomenology, lack of reliable molecular markers of the suppressor cell population, and conflicting data; yet, studies by Nishizuka and colleagues showed that mice thymectomized at 3 days after birth achieved systemic organ-specific autoimmunity (3). Critically, these diseases could be prevented or even reversed with the adoptive transfer of CD4^+^CD25^+^ T cells (4). This was reminiscent of the Medawar work demonstrating the importance of tolerance induction early in life and implicated a small subset of T cells in the biology.

In the late 1990s, studies of a mouse strain with multi-system autoimmunity (*scurfy* mice) led to the identification of the transcription factor Foxp3 (5). The *scurfy* mouse, which was originally developed at Oak Ridge National Labs during the Manhattan (atomic bomb) project, displayed many of the same attributes of the disease seen in neonatally thymectomized mice (6). Furthermore, a disease in male children—immune-mediated, polyendocrinopathy, X-linked—resembled the pathology found in *scurfy* mice, and it was shown that these patients also possessed mutations in FOXP3 (7). Further studies by multiple groups demonstrated that FOXP3 is expressed primarily in the CD4^+^CD25^+^ subset of T cells that were shown to be capable of controlling autoimmunity in mice (8–10). Thus, by the mid-2000s, T regulatory (Treg) cells could be defined at the molecular level in both mice and humans, and their critical role in maintaining immunological tolerance was unequivocally established.

Over the last 20 years, immunological tolerance has been linked to multiple mechanisms (1, 11). However, thymically derived Treg cells represent the dominant mechanism for maintaining normal immune homeostasis and preventing fatal multi-organ autoimmunity. These cells possess a broad repertoire of self-reactivity and are found in every tissue of the body. Preclinical studies have demonstrated that Treg cells from the periphery can traffic to many tissues and can treat many different models of autoimmune disease, and their persistence within the tissue depends on the presence of self-antigens and commensal bacteria (12–14). Importantly, there is strong evidence that the local tissue and the infiltrating Tregs have a selective activity for proteins present in the specific inflamed tissue and, in some instances, the local microbiota (15–17). While Treg cells from the blood are primarily naïve-like cells, tissue-resident Tregs are “tuned” to the specific environment within a given tissue. They can display different chemokine receptors, transcription factors, and metabolic properties.

In addition to the dominant population of thymically derived FOXP3^+^ Treg cells, in the periphery, factors such as TGFβ can convert conventional T cells to FOXP3^+^ Tregs, especially in the gut (18). Such peripherally “induced” treg cells are not as clearly defined at the molecular level as the thymically derived FOXP3^+^ Treg cells, and in pre-clinical models, they do not appear to be as stable (19, 20). In this regard, Dijke et al. performed an examination using discarded human thymuses as a source of therapeutic Tregs. They concluded that thymic Tregs are superior to Tregs derived from peripheral or cord blood (21).

There are multiple mechanisms by which Treg cells have been shown to inhibit immune function (22, 23). Tregs directly suppress antigen presentation directly through the engagement of checkpoint receptors, CTLA-4, and stripping major histocompatibility complex proteins of the cell surface of antigen-presenting cells (24). In addition, Tregs function indirectly and mediate bystander suppression through the production of suppressive cytokines (including IL-10, IL-35, and TGFβ), consumption of T cell growth factors (including IL-2), and altering the local metabolic environment to limit Teff cell activity (11). It is important to note that Treg activation is dependent on activation through the T cell receptor and CD28 engagement as a co-stimulatory signal. In fact, the ability to respond to IL-2 is also TCR/CD28 dependent, supporting a critical interplay of these signaling pathways in determining the ability of these cells to mediate bystander suppression (25–27). Finally, Tregs have also been shown to mediate what is called infectious tolerance (28–31). Gershon and Kondo were the first to coin the phrase “infectious tolerance” in the 1970s (2, 28). The term was based on the observation, by this group and others (29–31), that long-term tolerance to allogeneic skin grafts and other immune settings was induced by adoptively transferred Tregs but, once established, remained durable by the recruitment and development of other immunosuppressive cell populations. This ability of Tregs to promote other regulatory cells is likely to provide more robust and durable tolerance. In addition to inhibition of inflammation, Treg cells also promote the repair of damage by releasing tissue and stem cell factors (32, 33). The multifaceted activity of Treg cells provides a means to reduce inflammation with one cell type but *via* many different mechanisms, and thus Treg and Treg-friendly immune therapies have therapeutic potential in a variety of autoimmune, inflammatory, and transplant-related diseases.

One major concern with any potential tolerogenic therapy is the durability of the treatment. For most therapies, patients must take their medications on a daily, weekly, or monthly basis. In many instances, the inflammatory mechanisms can become resistant to the pathway modulators, leading to a continued reduction in original efficacy. Living drugs, such as Treg cell therapies, hold the prospect of a one-and-done therapy due to the potential of the drug to be long-lived—for instance, in the case of CAR-T cell therapies in cancer, June and colleagues have shown that the cells can survive for more than 10 years (34). However, it is yet to be shown that Tregs have the same longevity, that is, where infectious tolerance may be a distinct advantage. Several pre-clinical studies have shown that one consequence of the multifunctional activity of Tregs is their ability to recruit and influence other immune cells (such as myeloid and naive T cells) to differentiate into regulatory cell populations (MDSCs, Tr1 cells, and additional Tregs), leading to an amplification of the immunoregulation such that the long-term effects of Treg therapy can be durable. In the end, the depth and the breadth of the therapeutic effect of Tregs will be crucial as new modalities of treatments for people with chronic and debilitating diseases. These therapies must, by necessity, lead ultimately to more than a modest step-wise improvement to the standard of care. In addition, a combination of precision medicine using biomarkers to identify specific patient segments that are most likely to respond to the therapy, combined with an ability to take advantage of the multiple immune regulatory activities or even manipulate the cells to maximize the therapeutic impact, will be required to fulfil the promise of these new therapies.

## 2 The tolerogenic potential of Treg therapies

Treg cells provide unique opportunities for meaningful therapeutic differentiation that can be envisioned to tackle the complex pathobiologies that underlie most autoimmune and inflammatory diseases. In contrast to other approaches, Treg therapies have been shown to induce immune tolerance, which means the ability to treat for a short period of time resulting in long-term disease-free existence. Moreover, Tregs can exert their activities in both lymphoid and non-lymphoid tissues due to the multiple mechanisms that the cells use to control the immunological responses (35). Indeed, in a variety of preclinical studies, Treg adoptive immunotherapy has been shown to effectively ameliorate systemic inflammation and specific organ injury. These model systems include a wide variety of autoimmune models, including inflammatory bowel diseases (36), type 1 diabetes (T1D) (37), experimental autoimmune encephalomyelitis (EAE) (38), collagen-induced arthritis (39), as well as organ transplant rejection (40) and non-immune diseases, such as stroke (41) and amyotrophic lateral sclerosis (ALS) (42), where inflammation plays a role in disease pathogenesis. Thus, Treg therapies have the potential to significantly improve a variety of autoimmune and inflammatory disease activities given the broad applicability of the therapeutic approach.

However, given the bystander properties of Tregs and the recognition that organ-specific Tregs play a critical role in controlling autoimmunity in the local tissue environment, it was hypothesized that Tregs with single-antigen specificities might have an increased specific activity and a greater safety profile. In fact, data in multiple autoimmune (43) and transplant settings (44) showed that Treg cells expressing a single T cell receptor (TCR) suppressed autoreactive, multi-specific Teff cells and led to long-term tolerance. In fact, preliminary data in humans suggest that alloantigen-specific Treg cells can be effective in the transplantation setting (45). More recent studies have utilized new synthetic biology approaches to provide singular antigen specificity to Tregs, including the introduction of specific TCR (46) and chimeric antigen receptors (CAR) (47) to target specific inflamed tissues. These studies have shown not only efficacy in multiple pre-clinical models but also 10–25-fold increased activity when compared with polyclonal Treg populations. Thus, given the polypharmacy nature of Tregs, their activity in mechanistically different diseases, and their ability to be engineered, Treg therapeutics represent a new approach to creating deep and durable treatments.

## 3 Challenges and key features to be addressed in developing Treg therapeutics

### 3.1 Will Treg therapy promote broad immunosuppression?

The advantage of CAR-engineered Treg therapy is that it is directed to a single antigen that is not dependent on HLA presentation and thus can be focused on antigens expressed in inflamed tissues. In contrast, TCR-engineered Treg cells engage HLA peptide-expressing cells which can be expressed in both the draining lymph node and the inflammatory sites. Although there are concerns that broad immunosuppression might occur in the presence of an acute infection or even in the context of cancer, healthy individuals routinely combat pathogens and cancer in the presence of antigen-specific Treg activity. There are examples where the deletion of Tregs in pre-clinical models can improve effector immunity, but, in general, Tregs are part of the homeostatic balance of a healthy immune response (48, 49). Data in the NOD mouse model of T1D, in which Treg therapy prevents the autoimmune destruction of pancreatic beta cells, demonstrate that viral immunity is preserved. In adoptive Treg therapy for clinical bone marrow transplant, immunity toward the cancer is likewise maintained. In fact, studies have shown that Treg activity at sites of pathogenic infection can limit the overall tissue damage, and uncontrolled chronic inflammation can be a driver for cancer in several settings (50). Finally, in recent years, low-dose IL-2 and IL-2 muteins have been tested as therapeutics in a variety of clinical settings given their ability to expand Tregs *in vivo* (51). In many of these studies, there has been two- to fivefold increases in Treg numbers in the circulation, even over a long-term treatment regimen, yet there have been no reported increases in pathogen infections or cancer, suggesting that Tregs are not overly immunosuppressive in the context of infections. Finally, as discussed above, Tregs need to constantly engage antigens to survive—that is, without ongoing TCR and CD28 engagement, Tregs fail to persist. Thus, the current preclinical and human data suggest that Treg therapy does not inhibit the natural immunity to pathogens or cancer.

However, despite the early results suggesting that Tregs will be a safe therapy, the potential liability of a broad anti-inflammatory activity is also being addressed in a variety of ways. First, several of the companies are incorporating tags and kill switches in therapeutic Treg products that might allow the patient to be treated with clinically approved monoclonal antibodies and small molecules to eliminate the adoptively transferred Treg. Additionally, efforts are underway to create regulated CARs and TCRs that allow the specificity to be modulated in case of adverse effects of the treatment. In addition, as in the CAR-T space in cancer, the use of mRNA or short-lived DNA to deliver the specificity will lead to a transient expression of the CAR or TCR. This might be especially useful in conditions in which a severe pathology is driven in a large part by inflammation and, once resolved, does not require ongoing Treg persistence. Examples include acute respiratory distress syndrome (ARDS) or stroke, among others. Such settings would benefit from or perhaps require the development of an off-the-shelf Treg cell product that might be short-lived.

### 3.2 What will be the potential adverse events following Treg therapies?

Assuming that the immunosuppressive activity of Tregs can be harnessed effectively to inhibit a disease-specific inflammation, several other concerns must be addressed in considering a robust cell therapy. Most importantly, the Tregs must maintain their phenotype and function stably over time, even in the harshest inflammatory settings. There are some studies of mouse Tregs showing that, under certain inflammatory conditions, a subset of Treg cells can lose Foxp3 expression and produce proinflammatory cytokines (52, 53). However, other studies suggest that Tregs are quite stable (54). Similar conflicting results have been seen in patients with autoimmune diseases, usually at the site of inflammation. This has raised concerns related to cytokine release syndrome (CRS) as seen in some cancer cell therapy settings using CAR-T cells. However, in these settings, CRS primarily occurs in the context of hematological malignancies that carry large antigen loads within the blood. Moreover, in many settings (such as transplantation and autoimmunity), the patients are on other immunosuppressive drugs known to moderate the effector cytokine production. In fact, the human clinical data suggests that Treg therapy does not promote CRS in a fashion like that of CAR Teff cells. The stability of Tregs is maintained, and Treg function has been shown to reduce inflammation and have less infection events compared with immunosuppression. That said, as highlighted below, there are multiple academic and company efforts to ensure the stability of these cells (**Table 1**).

**Table 1:** Companies involved in regulatory cell adoptive immunotherapy.

Company	Cell type	Cell source	Modification	Lead indication
Sonoma Biotherapeutics	FOXP3+ Treg cells	Autologous peripheral blood	Citp-CAR	RA
PolTREG	FOXP3+ Treg cells	Autologous peripheral blood	MOG-CAR	T1D
Quell Therapeutics	FOXP3+ Treg cells	Autologous peripheral blood	FOXP3 and HLA-A2-CAR	Liver transplant
Abata Therapeutics	FOXP3+ Treg cells	Autologous peripheral blood	MBP-TCR	MS
Sangamo Therapeutics	FOXP3+ Treg cells	Autologous peripheral blood	HLA-A2-CAR	Kidney transplant
Coya Therapeutics	FOXP3+ Treg cells	Autologous peripheral blood	NONE	ALS
TeraImmune	FOXP3+ Treg cells	Autologous peripheral blood	FVIII-TCR	Hemophilia
Tract Therapeutics	FOXP3+ Treg cells	Autologous peripheral blood	NONE	Kidney transplant
Cellenkos	FOXP3+ Treg cells	Allogeneic cord blood	NONE	ARDS
GentiBio	CD4+ T cells	Autologous peripheral blood	FOXP3 and islet-specific TCR	T1D
Tr1x	Tr1 cells	Adult peripheral blood	Undisclosed	Undisclosed
Cabaletta	Peripheral blood T cells	Autologous and Allogeneic T cells	CD19-CAR	Pemphigus vulgaris
Kyverna	Peripheral blood T cells	Autologous and Allogeneic peripheral blood T cells	regulated CD19-CAR	B cell autoimmunity
Atara	Peripheral blood T cells	Adult peripheral blood	EBV-reactive T cells	PPMS
ORCA Bio	FOXP3+ Treg cells	Allogeneic peripheral blood	NONE	GvHD

## 4 Lessons learned from clinical trials

Over the last 15 years, there have been more than 25 early-stage clinical trials conducted, mostly in an academic setting, to investigate the safety and biological activity of Tregs in diseases ranging from autoimmunity, organ transplantation, graft *versus* host, and other inflammatory diseases. An overall summary of a small “subset” of clinical trials is found in **Table 2**. These trials have led to clear evidence that the therapy is feasible, can be administered safely, and is tolerated for a long term. To date, there have been no life-threatening adverse events, including no observed increase in infections or evidence of cancer development (either the administered cells or in the patients). In fact, as confidence in the therapy has grown, more than 10 companies have initiated Treg programs using both conventional and engineered Treg cells. The first industry-sponsored trials are underway, with efficacy studies on the horizon, to assess the therapeutic potential of these novel cell therapies. That said, there are key lessons that have been learned from past studies that inform future efforts and have created potential opportunities to improve the therapies.

**Table 2:** Summary of a subset of clinical trials with Tregs in multiple diseases.

Study ID (www.clinicaltrials.com)	Study title (sponsor)	Phase	Product	Study status	Reference number
Autoimmune Diseases					
NCT01210664	T1DM immunotherapy using CD4^+^CD127^lo/−^CD25^+^ polyclonal Tregs (University of California San Francisco)	1	Expanded autologous CD4^+^CD127^lo/−^CD25^+^ polyclonal Treg	Completed	(55)
NCT02428309	Autologous polyclonal Tregs for lupus (University of California San Francisco)	1	Expanded autologous CD4^+^CD127^lo/−^CD25^+^ polyclonal Treg	Completed	(56)
NCT02691247	Safety and Efficacy of CLBS03 in adolescents with recent onset type 1 diabetes (The Sanford Project T-Rex Study - Lisata Therapeutics, Inc.)	2	Expanded autologous CD4^+^CD127^lo/−^CD25^+^ polyclonal Treg	Completed	(57)
NCT02772679	T1DM immunotherapy using polyclonal Tregs + IL-2 (TILT) (University of California San Francisco)	1	Expanded autologous CD4^+^CD127^lo/−^CD25^+^ polyclonal Treg	Completed	(58)
NCT02932826	Safety study and therapeutic effects of umbilical cord blood Treg on autoimmune diabetes (Second Xiangya Hospital of Central South University)	1/2	Expanded umbilical cord Tregs CD25^+^ CD4^+^ T cells	Active	NA
NCT03185000	Safety and efficacy of antigen-specific regulatory T cell therapy for patients with refractory Crohn’s disease (King’s College London)	1/2	Expanded autologous CD4^+^CD127^lo/−^CD45RA^+/^CD25^+^ polyclonal Treg	Completed	(59, 60)
NCT03239470	Polyclonal regulatory T cells (PolyTregs) for pemphigus (University of California San Francisco)	1	Expanded autologous CD4^+^CD127^lo/−^CD25^+^ polyclonal Treg	Completed	NA
NCT04691232	Autologous *ex vivo* expanded regulatory T (University of Erlangen-Nürnberg Medical School)	1	Expanded autologous CD25^+^ CD4^+^ plus rapamycin	Active	(61)
ISRCTN06128462	Cellular therapy of type 1 diabetes with T regulatory cells (Medical University of Gdansk)	1	Expanded autologous CD4^+^CD127^lo/−^CD25^+^ polyclonal Treg	Completed	(62)
Islet transplantation for type 1 diabetes					
NCT03444064	PolyTreg immunotherapy in islet transplantation (University of Alberta)	1	Expanded autologous CD4^+^CD127^lo/−^CD25^+^ polyclonal Treg	Completed	NA*
NCT04820270	Infusion of autologous T regulatory cells (Treg) at the time of treatment of allogenic islets (The Nordic Network For Clinical Islet Transplantation)	NA	Enriched autologous, CD4^+^CD127^lo/−^CD25^+^ polyclonal Treg (No expansion)	Active	NA
NCT05349591	Cryopreserved polyclonal regulatory T cell immunotherapy in islet transplantation (University of Alberta)	1	Cryopreserved expanded autologous CD4^+^CD127^lo/−^CD25^+^ polyclonal Treg	Active	NA
Kidney transplantation					
NCT01446484	Treatment of children with kidney transplants by Injection of CD4^+^CD25^+^FoxP3^+^ T cells to prevent organ rejection (Pirogov Russian National Research Medical University)	1/2	Expanded autologous CD4^+^CD127^lo/−^CD25^+^ Polyclonal Treg with/without Campath	Active	NA
NCT02088931	Treg adoptive therapy for Subclinical Inflammation in Kidney Transplantation (TASK) (University of California San Francisco)	1	Expanded autologous CD4^+^CD127^lo/−^CD25^+^ FOXP3^+^ Tregs	Completed	(63)
NCT02091232	Infusion of T-regulatory cells in kidney transplant recipients (The ONE Study) (Massachusetts General Hospital)	1	Expanded autologous CD4^+^CD127^lo/−^CD25^+^ FOXP3^+^ Tregs	Completed	(64)
NCT02129881	The ONE Study (UK Treg Trial) (Guy’s and St Thomas’ NHS Foundation Trust)	1	Expanded autologous regulatory T cell product	Completed	(64)
NCT02145325	Trial of Adoptive Immunotherapy with TRACT to Prevent Rejection in Living Donor Kidney Transplant Recipients (TRACT) (Northwestern University)	1	Expanded autologous CD4^+^CD127^lo/−^CD25^+^ FOXP3^+^ Tregs	Completed	(65)
NCT02244801	Donor-alloantigen-reactive regulatory T cell (darTreg) therapy in renal transplantation (The ONE Study) (University of California San Francisco)	1	Expanded autologous alloantigen-specific CD4^+^CD25^+^FOXP3^+^ Tregs	Completed	(64)
NCT02371434	The ONE Study trial (Charité University)	1/2	Expanded autologous polyclonal CD4^+^CD25^+^FOXP3^+^ polyclonal Tregs	Completed	(64, 66)
NCT02711826	Treg Therapy in Subclinical Inflammation in Kidney Transplantation (TASK) (University of California San Francisco)	1/2	Expanded autologous CD4^+^CD127^lo/−^CD25^+^ FOXP3^+^ polyclonal Tregs	Completed	NA
NCT03284242	A pilot study using autologous regulatory T Cell Infusion Zortress (everolimus) in renal transplant recipients (University of Kentucky)	1	Expanded autologous CD4^+^CD127^lo/−^CD25^+^ FOXP3^+^ Tregs + Everolimus	Active	NA
NCT03867617	Expanded autologous regulatory T cell therapy and tocilizumab together with donor BM infusion in HLA-mismatched living donor kidney transplant recipients (Medical University of Vienna)	1/2	Expanded autologous regulatory T cell therapy, tocilizumab, and donor bone marrow	Active	NA
NCT03943238	TLI, TBI, ATG, and hematopoietic stem cell transplantation and recipient Tregs therapy in living donor kidney transplantation (Stanford University)	1	Expanded autologous Tregs	Active	NA
NCT04817774	Safety and tolerability study of chimeric antigen receptor T-reg cell therapy in living donor renal transplant recipients (STeadfast) (Sangamo Therapeutics)	1/2	HLA-A2 CAR CD4^+^/CD45RA^+^/CD25^+^/CD127^lo/-^	Active	NA
ISRCTN 11038572	TWO study: cell therapy trial in renal transplantation (University of Oxford)	2	Expanded autologous. polyclonal Tregs	Active	(67)
Liver transplantation and other treatments					
NCT01624077	Safety study of using regulatory T cells induce liver transplantation tolerance (Treg) (Nanjing Medical University)	1	Autologous Treg cells from thymic tissue (thyTreg), CD25+ Foxp3+ cells	Unknown	NA
NCT02166177	Safety and Efficacy Study of Regulatory T Cell Therapy in Liver Transplant Patients (ThRIL) (Guy’s and St Thomas’ NHS Foundation Trust)	1	Expanded autologous regulatory T cell product	Completed	(68)
NCT02188719	Donor-alloantigen- reactive regulatory T Cell (darTregs) in Liver Transplantation (deLTa) (NIAID/University of California San Francisco)	1/2	Expanded autologous donor-alloantigen-reactive Tregs (darTregs)	Terminated	NA
NCT02474199	Donor alloantigen reactive Tregs (darTregs) for calcineurin inhibitor (CNI) reduction (ARTEMIS) (NIAID/University of California San Francisco)	1/2	Alloantigen-expanded autologous CD4^+^CD127^lo/−^CD25^+^ Treg cells	Completed	(69)
NCT03577431	Liver transplantation with Tregs (LITTMUS MGH) (NIAID/Mass Gen Hosp)	1/2	Expanded autologous donor alloantigen-reactive CD4^+^CD127^lo/−^CD25^+^ Treg cells (arTregs)	Terminated	NA
NCT03654040	Liver transplantation with Tregs (LITTMUS-UCSF) (NIAID/University of California San Francisco)	1/2	Expanded autologous donor alloantigen-reactive CD4^+^CD127^lo/−^CD25^+^ Treg cells (arTregs)	Terminated	NA
NCT04924491	Cell Therapy With Treg Cells Obtained From Thymic Tissue (thyTreg) to Prevent Rejection in Heart Transplant in Children (Hospital General Universitario Gregorio Marañon)	1	Thymic tissue (thyTreg)-derived CD25+ Foxp3+ cells	Active	(70)
NCT05234190	Safety and clinical activity of QEL-001 in A2-mismatch liver transplant patients (LIBERATE) (Quell Therapeutics Limited)	1/2	HLA-A2 CAR-Treg	Active	NA
UMIN-000015789	Tolerance induction by a regulatory T cell-based cell therapy in living donor liver transplantation (Masonic Cancer Center, University of Minnesota)	1/2	Donor-reactive Treg-enriched cell product	Active	NA
Graft versus host disease					
NCT00602693	T-Regulatory Cell Infusion Post Umbilical Cord Blood Transplant in Patients With Advanced Hematologic Cancer (Masonic Cancer Center, University of Minnesota)	1	Umbilical cord blood expanded CD4^+^CD25^+^ Tregs	Completed	(71)
NCT01634217	First-in-human phase 1 trial of induced Treg cells for graft-versus-host disease prophylaxis in HLA-matched siblings (Masonic Cancer Center, University of Minnesota)	1	Human CD4^+^25^-^ T cells cultured in IL-2, rapamycin, and transforming growth factor β (TGFβ) along with anti-CD3-loaded artificial antigen-presenting cells	Completed	(72)
NCT01660607	Phase 1-2 MAHCT w/ T Cell Depleted Graft w/ Simultaneous Infusion Conventional and Regulatory T Cell (Stanford University)	1	Donor regulatory T-cells	Completed	(73, 74)
NCT01795573	Ex-vivo Expanded Donor Regulatory T Cells for Prevention of Acute Graft-Versus-Host Disease (H. Lee Moffitt Cancer Center and Research Institute)	1	Expanded donor regulatory T-cells	Completed	(73)
NCT01903473	Donor regulatory T cell infusion in patients with chronic graft-*versus*-host disease (GVHD) (University of Liege)	1	CD4^+^CD25^+^FoxP3^+^ T cells + IL-2 + Rapa	Terminated	NA
NCT01937468	Trial of regulatory T-cells plus low-dose interleukin-2 for steroid-refractory chronic graft-*versus*-host disease (Dana-Farber Cancer Institute)	1	CD4^+^CD127^lo/−^CD25^+^ Treg + low dose IL-2	Active	NA
NCT02526329	Donor regulatory T cells in treating patients with visceral acute graft-*versus*-host disease after stem cell transplant (Dana-Farber Cancer Institute)	1	CD4^+^CD127^lo/−^CD25^+^ Treg cells	Suspended	NA
NCT02749084	Multiple donor Treg DLI for severe refractory chronic GVHD (TREG2015001) (Universitaria di Bologna)	1	Non-expanded purified Tregs (CD8 and CD29 negative, CD25^hi^ cells)	Completed	NA
NCT03683498	Donor regulatory T-cells for steroid-refractory chronic graft-*versus*-host disease (GVHD-TReG) (Fundación Pública Andaluza para la gestión de la Investigación en Sevilla)	1	Expanded donor regulatory T-cells	Completed	(75)
NCT04013685	A Study of Engineered Donor Grafts (TregGraft/Orca-T) in Recipients Undergoing Allogeneic Transplantation for Hematologic Malignancies (Orca Biosystems, Inc.)	1	Non-expanded purified Tregs CD4^+^CD127^lo/−^CD25^+^	Active	(76)
Other diseases					
NCT01087515	Pilot study for cell-based therapies in patients with asthma (Hannover Medical School)	1	Fresh Treg	Completed	NA
NCT03241784 NCT04055623	T-Regulatory Cells in Amyotrophic Lateral Sclerosis (The Methodist Hospital Research Institute)	1	CD4^+^CD25^+^FOXP3^+^ Tregs with low-dose IL-2 in ALS	Active	(77, 78)
NCT04468971	Regulatory T Cell infuSion fOr Lung Injury Due to COVID-19 PnEumonia (RESOLVE) (Cellenkos, Inc.)	1	Umbilical cord blood-derived Treg	Completed	(79)
NCT05695521	Regulatory T cells for amyotrophic lateral sclerosis (REGALS) (Cellenkos, Inc.)	1	Umbilical cord blood-derived Treg	Active	NA
NCT05027815	Tregs for the treatment of acute respiratory distress syndrome (ARDS) associated with COVID-19 (regARDS) (University of California San Francisco)	1	Cryopreserved *ex vivo* expanded polyclonal CD4^+^CD127^lo/-^CD25^+^ T regulatory cells	Terminated	NA

NA, not available.

### 4.1 Experiences in Treg expansion

Fundamental research on Tregs demonstrated that the cells signal in a similar fashion as conventional T cells, requiring both a TCR and CD28 co-stimulatory signals for maximal expansion. In addition, Tregs have been shown to be exquisitely dependent on the IL-2 growth factor for both expansion and survival. Moreover, Tregs coming from individuals with autoimmune or other inflammatory diseases can be dysfunctional, leading to differences in expansion capabilities and potential phenotype changes, such as increased interferon production, when compared with Treg derived from healthy individuals. Thus, investigators employed similar tools as those used for T conventional cell expansion, namely, the use of anti-CD3/anti-CD28-coated beads in combination with IL-2 to expand Tregs. In previously published studies, the cells are routinely expanded 300- to 500-fold in 14 days. The purity of the expanded Treg populations is usually over 90% FOXP3^+^, and high levels of multiple phenotypic and functional markers are expressed, including CTLA-4, GITR, LAP, and CD25 (55, 80).

Most importantly, the expanded Tregs have equal or better phenotypic and functional activity when compared with freshly isolated Tregs (55). This is especially evident in Tregs isolated from patients with autoimmune diseases, where genetic and/or environmental influences often result in Tregs that are less effective in multiple suppressive activities and, in many cases, less stable as read out by reduced FOXP3, CD25, and CTLA-4 expression as well as less ability to respond to growth factors such as IL-2. However, the process of expansion generally leads to a population expressing higher levels of key functional markers and “repair” of functional defects. In fact, in early studies of IL-2 therapies, the repair of endogenous Treg’s inability to respond effectively to IL-2 was not only reversed but found to be stable even a year after the discontinuation of cytokine therapy (81). Most importantly, the expansion of the Tregs derived from peripheral blood as well as cord blood was highly stable when examined over time after Treg transfer into patients—for instance, an analysis of circulating adoptively transferred Treg cells demonstrated the continued expression of key functional and phenotypic markers for at least a year post-transfer (55). The results of an extensive bulk and single-cell T cell receptor analysis suggest that the expanded population of Tregs remains broad after expansion. Thus, it is not surprising that the current efforts to develop Treg populations for therapy have begun to employ novel genetic approaches to homogenize and maximize Treg phenotype and functional activity. This includes the use of antigen-specific receptors, including CARs or TCRs, to focus the tissue specificity of the Treg product.

### 4.2 Experiences in Treg stability *in vitro* and *in vivo*

Despite the previously described limited animal studies, to date, there is no evidence of Treg instability in any human clinical studies. This result may be due to multiple reasons. First, the expansion process is much more robust in human *versus* mouse, and the likely preferential expansion of thymus-derived CD45RA naïve Tregs using current approaches may selectively result in the expansion of the most stable Treg populations. In fact, the literature suggests that the majority of “unstable” Tregs are likely derived from induced peripheral Tregs, generated in the periphery *in vivo* upon encounter with TGFβ and other Treg-promoting cytokines (19, 22). Moreover, *in vitro* studies suggest that human Tregs isolated and expanded under the conditions noted above are “resistant” to certain inflammatory cytokines suspected of promoting instability in rodent Tregs—for instance, culturing human Tregs with IL-6 or TNFα (both present in highly inflamed tissues) routinely results in increased expansion and function, which are likely due to the upregulation of the TNFR2 receptor on the expanded Tregs (52, 82, 83). Finally, it cannot be ruled out that unstable Tregs are only present in tissues subject to extreme inflammatory milieu; however, as will be noted below, in several clinical settings, biopsy analysis post-transfer showed that Tregs in target tissues expressed higher levels of FOXP3 and CTLA-4 at 3 months post-transfer of Treg. Thus, it is likely that current expansion technologies result in the production of highly purified Treg populations that are stable in disease settings (including autoimmune lesions and inflamed transplanted organs).

At present, the majority of Treg products generated for clinical application are autologous. For autoimmune diseases, this means that the expansion protocol needs to consider the possibility of contaminated activated Teff cells, which express high levels of CD25 like Tregs, as well as genetically and environmentally induced Treg dysfunction. One approach to avoid these potential issues has been to use allogeneic Tregs derived from healthy individuals or umbilical cord blood (UCB) (72, 79). UCB Tregs are overwhelmingly naïve and have a very broad repertoire (84). In some cases, such as in the suppression of graft *versus* host disease, this has the added advantage of 5%–15% of the Tregs having alloreactivity and thus recognize the class II MHC of the host. However, there are multiple risks associated with this approach. First, should the Treg cells become unstable in patients, there is an expanded repertoire that can target the allogeneic tissue, be it host or transplanted tissues. Secondly, the host immune system can recognize and destroy the adoptively transferred Treg product. This was seemingly apparent in the first cord blood-derived Treg study in GvHD, where the cell product had a shorter duration *in vivo* when compared with autologous cells in other settings (71, 72). However, there are circumstances where an off-the-shelf allogeneic product may be required, such as in the treatment of acute ARDS, such as COVID, where the cells need to be administered immediately (79). As will be highlighted elsewhere, new approaches to both genetically engineered Tregs as well as potential stem cell-derived Treg products may enable the disruption of core allogeneic proteins, such as MHC antigens, and avoid rejection. Interestingly, the inherent alloreactivity of Tregs has been exploited in the organ and islet transplant setting, where investigators have either relied on the resident alloreactive Tregs in the polyclonal Treg population or developed approaches to selectively expand the alloreactive cells prior to transfer. This approach has great potential but may be superseded by the genetic engineering approaches described below.

It should be noted that, in some settings, the challenge of high purity and stability has required additional precautions in the development of Treg products—for instance, Teff cell contamination has been an issue due to inadequate separation technology and/or patient cell surface phenotype variation. As noted previously, preclinical studies suggest that peripherally derived Tregs, especially Helios^-^ Tregs, are less stable than their thymus-derived counterparts. Thus, depending on the source of Tregs cells, the potential for Teff or Teff-like activity is possible. To avoid some of the challenges to isolating and expanding highly stable and pure Tregs, investigators have taken advantage of the selective ability of rapamycin to suppress effector T cell growth while preserving Treg expansion. The differential mechanistic role of mTORC1 and mTORC2 in Treg *versus* Teff cells results in the rapamycin-induced increased stability of Tregs, selectively inhibiting Teff cell expansion (85, 86). In the future, new genetic engineering approaches that are now being developed will both increase the ability to isolate highly pure populations of Tregs as well as provide safety switches and a more stable Treg population in settings where instability remains a risk.

### 4.3 Experiences in early phase 1/2 trials of Tregs

As noted in **Table 2**, there have been over 25 phase 1/2 clinical trials to test the efficacy of autologous and allogeneic polyclonal adult and UCB-derived Tregs in a variety of diseases. However, there have been only a limited number of presentations and no published phase 2 studies using polyclonal or antigen-specific engineered Tregs. In the case of the T1D study, NCT02691247, the company, Caladrius, released a press release saying that the trial did not meet its primary endpoint. In the case of the ALS study, recruitment was impacted by COVID-19, and no meaningful evaluation could be made. The early Treg studies were performed in patients experiencing acute or chronic GvHD, using either cord blood allogeneic or adult-derived autologous Tregs, based on supportive pre-clinical mouse data. In the first study reported out of Poland, *ex vivo*-expanded CD4^+^CD127^lo/-^CD25^+^ Treg cells were infused into patients with active GvHD (87). A similar study was performed with cord blood-derived Tregs by Wagner and colleagues (71, 72). The studies showed that pure Tregs could be isolated, expanded, and transferred into patients with the disease. It is also worth noting that one of the impetuses for using cord blood-derived Tregs was the greater ease in purifying the population due to the distinctly high levels of CD25 on these cells. However, although the typical expansion time—14 days—of adult-derived autologous Tregs is sufficient to generate target doses, in the case of cord blood, the small number of Treg at the start of culture required more extensive expansion, often three to four cycles (71, 72). This can lead to increased instability of the product and, in some cases, diversion to a Th2 (IL-4)-producing Treg functional state. In the cord blood-derived allogeneic Treg study, there was an early suggestion of efficacy. This approach in GvHD is now being examined, with or without low-dose IL-2, in a formal phase II study to determine the efficacy and biomarker activity.

### 4.4 Autologous Tregs have been tested in a variety of clinical settings

The first clinical studies of autologous Tregs in autoimmunity were conducted in patients with T1D. These studies included children and adults recently diagnosed with the disease and were based on extensive preclinical data that suggested that Tregs were able to reverse diabetes in a spontaneous animal model of T1D, the NOD mouse, if given soon after the diagnosis. The protocol for Treg therapy in T1D patients required the transfer of *ex vivo*-expanded autologous polyclonal CD4^+^CD127^lo/-^CD25^+^ Treg cells within 6 weeks of diagnosis (55, 58, 62). In these studies, the c-peptide levels, as a readout of insulin production, were monitored for up to 2 years.

While there was a suggestion of efficacy, the studies were small and uncontrolled. In the study conducted in Poland, the treated children had higher c-peptide levels and lower insulin requirements than the historical data in untreated children (62). In the first phase I clinical trial conducted in the United States, *ex vivo*-expanded polyclonal Treg cells were infused into 14 patients with new-onset T1D in doses ranging from 5 × 10^6^ to 2.6 × 10^9^ cells. The infusions were well tolerated, and the c-peptide levels in most patients remained stable for 1 year, although efficacy could not be conclusively shown (55). Notably, the cell pharmacokinetic analysis of Treg cells labeled with deuterium during *ex vivo* expansion showed that a subset of the infused Treg cells persisted in peripheral blood for at least 1 year and that the Treg cells remained phenotypically stable after the infusion. A phase II clinical trial performed by Caladrius Biosciences, in which 113 newly diagnosed (less than 100 days post-T1D diagnosis) adolescents with T1D were either untreated or infused with autologous *ex vivo* polyclonally expanded Treg cells (56). The treated group failed to show a clinical effect of the therapy, *i.e.*, preservation of c-peptide production 1 year after the infusion. While the highly enrolled study was found to be safe, with no major serious adverse events reported, the study highlighted the need to consider a more targeted antigen-specific Treg population to gain true efficacy as seen in the pre-clinical studies.

Recent years have seen an increased number of polyclonal Tregs trials in multiple different clinical settings. There have been multiple studies of autologous Treg therapy in patients receiving kidney, liver, and, most recently, islet transplantation (**Table 2**). Once again, the therapies have been deemed safe, and in several instances, there has been biomarker evidence (such as reduced effector cytokine production in the urine of patients treated at the time of protocol biopsy proven inflammation) suggesting biologic activity. One biomarker study, conducted in the liver transplant setting, showed that the therapy was safe, and there was an altered effector cytokine profile in the blood. Another example of a small safety trial was a study conducted by Appel and colleagues looking at the effect of Treg therapy in patients with ALS. The pre-clinical results had suggested that, even in this non-immune-mediated disease, the control of the associated inflammation could impact disease progression. In the study of Appel et al., three patients with highly advanced ALS were treated with multiple courses of autologous Tregs along with low-dose IL-2 and monitored for disease progression (77, 78). There were some suggestions that the disease progression slowed during immunotherapy; however, it was not clear what role the IL-2 was playing in the therapeutic effects.

One of the challenges in the early Treg interventional studies in autoimmunity has been the small number of patients enrolled in any given trial and the limited biomarker analyses. Although biopsies were possible in the transplant studies, any effects were difficult to interpret given the concomitant treatment of patients with multiple immune-suppressive drugs. Furthermore, in many settings, biopsies were not obtained due to logistical and safety reasons. However, several phase 1 studies in autoimmune settings have been performed where serial biopsies could be performed. In one published study, a patient with cutaneous lupus erythematosus was treated with 1 × 10^8^ *ex vivo*-expanded CD4^+^CD127^lo/-^CD25^+^ Treg cells. The affected skin was biopsied both on day 0 (prior to Treg injection) and at 12 weeks. The biopsy was assessed for changes in Tregs and Teff cells. As noted above, there was a significant increase in the number of Tregs when the 12-week biopsy was compared with that of day 0. More importantly, the level of FOXP3 was significantly increased in the cells isolated from the biopsy at week 12. In contrast, at the same time point, there was an approximately 75% reduction in interferon-producing CD4^+^ Teff cells when compared with the day 0 biopsy. Interestingly, in some patients, IL-17-producing T cells were preserved. Thus, a shift from IFNγ to IL-17 may lead to reduced inflammation and increased tissue repair. Similar results were observed in a phase 1 trial in patients with pemphigus (unpublished results/manuscript in preparation). In one patient with paired samples, the percentage of Tregs cells increased in the skin biopsy at week 12, similar to that observed in the lupus patient (56). There was a significant decrease in both the number of Teff cells and their ability to produce interferon at 12 weeks. These results together suggest that the Treg therapy had an impact on the microenvironment of the pathogenic lesion, resulting in diminished inflammatory processes.

There have been several studies looking at alloantigen-reactive Tregs based on preclinical efficacy studies. Overall, Treg cell infusions have been safe and well tolerated. A challenge in these studies, however, has been in manufacturing the cells, potentially because these patients are chronically immunosuppressed, which alters the Tregs both quantitatively and qualitatively. In addition, it appears that the alloreactive Tregs can be sequestered in the target tissue—in the cited instance, the liver—resulting in a reduced precursor frequency in the blood (69).

## 5 Opportunities to enhance Treg cell therapy

### 5.1 Enhanced antigen-specific activation

Controlling the local activation and proliferation of Tregs is key to maximize their therapeutic potential. Because Treg cells require engagement through the TCR/CD28 for Treg cell survival and regulatory function, harnessing these physiological signals is an attractive option for controlling Treg activation. TCR or CAR engineering has the potential to direct Treg activation in an antigen- or tissue-restricted way, thus leading to the control of Treg activation, and only in the tissues of particular interest. For TCR-Treg development, the identification of tissue-specific antigen/peptide targets is critical. With CD4+ restriction, class II peptides are the most obvious choice, but the tools to identify and characterize class II-restricted peptides fall far behind those of class I. For class I-restricted TCRs, it is possible to isolate receptors that are independent of CD8 co-receptor activity or the Tregs can be genetically modified to express CD8 which would allow MHC class I-restricted antigens to become viable targets for TCR-engineered Tregs. From an engineering perspective, endogenous TCRs can be left intact if engineered TCRs are still able to preferentially pair and out compete endogenous receptor complexes for TCR signaling components needed to transduce the signals required. Forcing a very high level of expression of the engineered TCRs on the cell surface, utilizing known mutations to induce preferential chain pairing, capturing, or tethering them to the cell surface, and enhancing recycling are all viable methods being explored in the field.

While using TCRs for Treg recognition is appropriate in many settings—particularly those with a strong HLA association, another approach is the use of chimeric antigen receptors (CARs) which can avoid HLA restriction completely. One advantage is that the CAR affinities can be finely tuned to change both the level of antigen-specific Treg activation as well as the amount of off-target “tonic” signaling. Moreover, the CARs can be engineered to alter the site and timing of activation. Whereas TCR-restricted specificities would need cell–cell contact, CAR activation can also be driven by soluble factors, tissue matrix, or exogenous ligands. Advances in high throughput binder generation with exquisite specificity and selectivity have enabled the generation of novel CARs with confirmational epitopes, overcoming some of the challenges to the identification of tissues and disease-specific reactivities. CARs can be re-engineered to have TCR-like properties and even recognize specific peptide/MHC complexes, bypassing the need for TCR engineering while capturing the natural site of Treg activation at the cell surface of antigen-presenting cells (88).

### 5.2 Enhancing efficacy

Most new anti-inflammatory drugs fail because of two main reasons: lack of clinical safety and lack of clinical efficacy. If the early clinical work with polyclonal Tregs continues to be a guide, Treg therapies are likely to be safe. As such, the lack of clinical efficacy remains the biggest unknown in the field today. Harnessing the natural homing and tissue-specific regulatory mechanisms of Tregs as well as engineering new mechanisms of immune regulation may be the key to achieving a meaningful and durable clinical effect. Here are some key areas of focus for the field.

#### 5.2.1 Cytokines

As potent immune regulators, cytokines are particularly important controllers of Treg-mediated effects and provide many opportunities for engineered products. Enhancing or stabilizing the secretion of anti-inflammatory cytokines (such as IL-10 and TGFβ) may, in certain indications, enhance the anti-inflammatory actions and better control the magnitude of tissue damage. Tregs could also be engineered to express dominant negative or decoy receptors (cell surface or secreted) negating the effects of local and systemic pro-inflammatory cytokines. The remarkable ability of Tregs to home to sites of inflammation could also be harnessed to deliver locally acting anti-inflammatory molecules to the right cell at the right time. This may give new life to dozens of therapies whose off-tissue pharmacology precluded further clinical development. Tregs could also be engineered to respond to certain cytokine stimuli in new ways—for instance, there may be an opportunity to control Treg activation, persistence, and responsiveness to local inflammatory signals. Logic gated systems have gained popularity in the CAR-T effector space in the past few years by harnessing tissue-specific signals (including cytokines) to turn on (or shut down) proliferation or effector functions (89, 90).

#### 5.2.2 Tissue-protective and repair factors

In addition to the well-documented anti-inflammatory effects of Tregs, it is becoming increasingly clear just how important these cells can be in maintaining tissue homeostasis (32, 33)—for example, amphiregulin (shown to be secreted by Tregs) can halt tissue damage and encourage tissue repair and the reestablishment of normal tissue function. Creating Tregs capable of producing or enhancing the natural tissue-protective and repair factors within a specific tissue will increase the potential that Treg therapies will lead to transformative, durable treatments—for example, logic gated systems could be used to “switch” Treg cells from an anti-inflammatory function to a repair function once inflammation has subsided.

##### 5.2.2.1 Homing

As mentioned above, Treg cells have the capacity to enter most tissues of the body, but they most efficiently enter sites of inflammation. In some instances, this may lead to unwanted off-disease effects and/or reduce the number of transferred cells homing to important sites of action. To induce tissue homing bias, it is possible to select for unique tissue homing subsets or to engineer homing capabilities to bulk populations of Tregs to send them to specific locations—for example, in treating inflammatory bowel diseases, it may be advantageous to select for α4β7-expressing Tregs capable of homing to MAdCAM-1-expressing endothelium in sites of tissue inflammation and in Peyer’s patches and mesenteric lymph nodes (91). This may enhance the total tissue load of Treg and enhance both the anti-inflammatory and the pro-repair mechanisms. Alternatively, one could select against or modify homing receptors that aid the transit of Tregs to other tissues and sites of cell clearance—for example, in the liver (a major site of T cell clearance). Homing receptors are interesting targets for logic gated systems as described above. A homing receptor-mediated logic gate could be used to selectively kill cells homing to unwanted sites of action (for example, the CNS) and avoid any consequences of tissue-specific immunosuppression in sites of ongoing infection or increased tumor risk. In this regard, Hoeppli et al. demonstrated that the migration capacity of human Tregs can be tailored by adding cytokines (IL-12 and IFNγ) and or/metabolites (retinoic acid) to culture conditions during *in vitro* expansion (92).

### 5.3 Durability and stability

#### 5.3.1 Enhanced growth factor independence

As noted above, Treg cells are highly dependent on growth factors for survival, activation, and proliferation. Although many cytokines and growth factors can influence Tregs, IL-2 is a critical target for advanced genetic engineering. As discussed above, in proinflammatory conditions, particularly those mediated by T cells, IL-2 is in abundance, and Treg are readily able to harness IL-2 through their high-affinity IL-2 receptor complexes. This has a twofold effect: it stimulates Treg expansion (and function) while capturing IL-2 and thus prevent Teff cells in the environment from receiving a needed growth factor, leading to activation-induced cell death. In those indications where there is insufficient IL-2—for example, in non-T cell driven inflammation—it may be necessary to provide IL-2 signals directly. As noted previously, in the ALS clinical trials, exogenous IL-2 was provided as support for expanded Treg cells following infusion. Tregs may be engineered to provide a cell-intrinsic IL-2 signal in several ways, including IL-2 secretion, IL-2 tethering, and modulation of intracellular signaling pathways. Given the central importance of IL-2 signaling in Tregs, coupled with the inconsistent presence of IL-2 in inflammatory disease of interest, it is likely that IL-2 will remain a key focus for Treg growth and persistence.

#### 5.3.2 Enhancing stability

Although data surrounding the plasticity of adoptively transferred human Tregs is lacking, in certain disease settings, Tregs can become destabilized (as shown by changes in FOXP3 expression and genome methylation status). As Treg therapies utilize TCRs and CARs to confer disease and/or tissue specificity, ensuring the stability of any Treg product becomes paramount. As noted previously, existing clinical trials, even those using alloantigen-specific expanded Treg cells, have proven safe without evidence of a destabilized Treg product. Nonetheless, due to the lack of relevant long-term model systems for using human Treg cells, there is considerable interest in maximizing Treg product stability. There are several approaches to further enhance Treg stability, ranging from overt over-expression of FOXP3 to modification of endogenous genomic loci. One such approach is epigenetic modification of the FOXP3 locus to stabilize Treg phenotype and functional activity (93). By targeting the CAR or TCR specificity to the FOXP3 locus, the stability of the Tregs would be linked to the expression of an introduced TCR or CAR. Other pathways (including IL-2 modulation) may also be effective at promoting FOXP3 expression and lineage stability. In all cases, stability will need to be assessed in the context of a pro-inflammatory environment.

#### 5.3.3 Allogenic Treg products

There is an increasing interest in the use of allogenic cell products, including gene-modified mature Tregs and iPSC-derived off-the-shelf cell platforms. Beyond the advantages from a cost-of-goods and deliverability perspective, a key advantage is the ability to ensure product consistency and limit patient-to-patient variability. Additionally, the ability to use healthy donor cells would eliminate any patient segment considerations on Treg quality or quantity. Finally, as noted earlier, an off-the-shelf product would be more amenable to rapid dosing for acute indications such as ARDS or stroke in which it would be impossible to make autologous Tregs quickly enough to impact pathology.

Another significant advantage to an allogeneic product, particularly based on iPSC technology, would be the ability to make several (maybe even dozens) of genetic modifications in a serial manner. It is conceivable to generate “panels” of engineered cells with, for example, differing specificities and then match a particular antigen-specific Treg with a patient segment. Each modification can be thoroughly characterized in isolation or in conjunction with other modifications before clinical studies. Allogenic products may be more amenable to repeat dosing as well as exploring combination therapies. Despite the promise of allogenic products, CAR-T effector clinical development is just beginning. Both engineering and validation have proven more difficult than expected.

## 6 Concluding remarks

Autoimmune disorders arise from defects in immune tolerance and affect more than 50 million people in the United States and more than 4% of the world’s population. Autoimmune disorders have a high impact on an individual’s morbidity and mortality as well as their quality of life, given that their chronicity, various organ manifestations, and association with co-morbidities are common and devastating for many sufferers. Despite several transformative medicines that have improved many autoimmune diseases, most patients do not adequately respond to existing therapies. Long-term drug therapy is required to maintain long-term efficacy; thus, there remains a major unmet medical need. Importantly, the goal of achieving true immune tolerance by re-establishing immune homeostasis remains unrealized.

As summarized in this review, Tregs, which constitute only a small percentage of circulating T cells, play a pivotal role in establishing and maintaining peripheral tolerance, preventing autoimmune diseases, and limiting chronic inflammatory diseases (94–97). In fact, Treg cells are now widely regarded as the primary cells involved in the persistence of peripheral tolerance. Their essential role is based on the cells’ multiple functionalities ranging from the production of key immunosuppressive cytokines and metabolites to the cell surface expression of key checkpoint molecules to control antigen presentation and metabolism. Importantly, the cells exhibit broad bystander suppression and can mediate infectious tolerance amplifying the impact of the cells, thus resulting in robust and durable efficacy.

As cell therapies have flourished in the cancer space as a new pillar of medicine, it is not surprising that Treg therapies, which have the capacity to control inflammation and autoimmunity, have become the latest approach to treat these devastating diseases. A wealth of pre-clinical data has shown that adoptive Treg cell therapy can be effective in the treatment of diseases ranging from autoimmunity and prevention of organ transplant rejection to cancer-related GvHD and neurological diseases such as ALS and stroke. Efforts are underway to exploit these cells as immunotherapies to treat and potentially prevent these diseases (97). Early clinical studies suggest that Treg adoptive immunotherapy is safe and can lead to biological and molecular changes that alter disease biology. In the oncology setting, however, it is likely that modifications to Treg cells may be required to fully achieve durable and long-lasting effects. Making use of synthetic biology, efforts are underway to genetically manipulate these cells to incorporate novel antigen specificities, altered cell characteristics including the reliance of certain growth factors and a carrier of payloads that can modify local inflamed tissues.

The future for exploiting Treg therapies will depend on solving key questions regarding process development, the assessment of Treg stability and durability, and the development of novel techniques to build new and enhanced activities. Although the field of Treg cell therapies is only at the “end of the beginning”, many have suggested that this living drug may finally enable achieving immune tolerance—the holy grail of immunotherapy.

## Author contributions

JAB, BM, JB and FR contributed to the writing, revision, reading, and approval of the submitted manuscript. All authors contributed to the article and approved the submitted version.
